# Dynamic and Differential Expression of Duplicated Cxcr4/Cxcl12 Genes Facilitates Antiviral Response in Hexaploid Gibel Carp

**DOI:** 10.3389/fimmu.2020.02176

**Published:** 2020-09-11

**Authors:** Wei-Jia Lu, Li Zhou, Fan-Xiang Gao, Yu-Lin Zhou, Zhi Li, Xiao-Juan Zhang, Yang Wang, Jian-Fang Gui

**Affiliations:** ^1^State Key Laboratory of Freshwater Ecology and Biotechnology, Institute of Hydrobiology, The Innovation Academy of Seed Design, Chinese Academy of Sciences, Graduate University of the Chinese Academy of Sciences, Wuhan, China; ^2^Institute of Marine Biology, College of Oceanography, Hohai University, Nanjing, China

**Keywords:** CXCR4, CXCL12, AMD3100, CaHV, antiviral response, innate immunity

## Abstract

Chemokine receptor *cxcr4* and its ligand *cxcl12* have evolved two paralogs in the teleost lineage. In this study, we have identified four duplicated *cxcr4* and *cxcl12* genes from hexaploid gibel carp, *Carassius gibelio*, respectively. *Cgcxcr4b*s and *Cgcxcl12a*s were dynamically and differentially expressed in immune-related tissues, and significantly up-regulated in head kidney and spleen after crucian carp herpesvirus (*Ca*HV) infection. Blocking Cxcr4/Cxcl12 axis by injecting AMD3100 brought more severe bleeding symptom and lower survival rate in *Ca*HV-infected fish. AMD3100 treatment also suppressed the up-regulation of key antiviral genes in head kidney and spleen, and resulted in more acute replication of *Ca*HV in *vivo*. Consistently, the similar suppression of up-regulated expression of key antiviral genes were also observed in CAB cells treated by AMD3100 after poly(I:C) stimulation. Finally, MAPK3 and JAK/STAT were identified as the possible pathways that *Cg*Cxcr4s and *Cg*Cxcl12s participate in to promote the antiviral response in *vitro*.

## Introduction

CXC chemokine receptor 4 (CXCR4) is an exclusive receptor of CXC chemokine ligand 12 (CXCL12), also known as stromal derived factor 1 (SDF-1) (1). CXCR4/CXCL12 axis plays a pivotal role in hematopoiesis, neurogenesis, germ cell development, vascular formation and inflammatory processes in mammals (2–6). During inflammation, CXCL12/CXCR4 axis controls the innate immune cell transporting between bone marrow and blood. CXCL12/CXCR4 interaction is responsible for the neutrophil retention in bone marrow, and both *cxcr4b* and *cxcl12* mutant can increase the neutrophil release into the bloodstream (5, 7, 8). As a weak neutrophil chemoattractant, CXCL12 can increase neutrophil migration to participate in the inflammatory response (9, 10). In mammals, CXCL12/CXCR4 axis diminishes the inflammatory reaction by increasing the production of inflammatory cytokine interferon-γ (IFN-γ) (11, 12), which forms a negative feedback control by decreasing the expression of CXCR4 (13, 14).

In teleost fishes, *cxcr4* and *cxcl12* also show high expression in immune tissues (15, 16). After virus or bacteria invasion, *cxcr4* and/or *cxcl12* are significantly up-regulated in Asian swamp eel (*Monopterus albus*) (17), rock bream (*Oplegnathus fasciatus*) (18, 19), orange-spotted grouper (*Epinephelus coioides*) (20, 21), freshwater carp (*Catla catla*) (22), and yellow croaker (*Larimichthys crocea*) (16). Therefore, *cxcr4* and *cxcl12* have been suggested as an important part in fish innate immune system (17–19). In zebrafish, Cxcl12a/Cxcr4b axis controls the neutrophil release into circulation, and antagonizes the wound-induced inflammatory signals (8, 23). In orange-spotted grouper, Cxcl12/Cxcr4 up-regulated their expression after nodavirus (NNV) infection, indicating their contribution to defensing the virus invasion (24). However, the specific role and underneath mechanism of Cxcr4/Cxcl12 axis in this process are still poorly understood in fish.

Gibel carp (*Carassius gibelio*) (3n = 6x = ~150 chromosomes) has been considered to undergo two extra polyploidy events (an early allopolyploidy followed by a recent autopolyploidy) in addition to the three rounds of whole genome duplication (WGD) shared by all teleost fishes (25–29). As a recurring polyploidy animal, it is an ideal system to investigate divergent expression and function of duplicated genes through WGD. Since 1980s, gibel carp has become an important aquaculture species in China (30). Along with the application of several improved varieties with excellent growth performance, such as allogynogenetic gibel carp “CAS III” (clone A^+^) and “CAS V” (clone F), the current annual production capacity has increased to about 3 million tons per year in China (31–33). However, due to the highly intensive culture, outbreak of epizootic crucian carp (*Carassius auratus*) herpesvirus (*Ca*HV) has led to enormous economic losses in main culture areas of Jiangsu province since 2012 (34–36). It is an urgent need to breed novel variety with *Ca*HV resistance. In our previous studies, IFN system genes and immunoglobulin genes were found to play vital roles in defensing *Ca*HV (32, 37, 38). Especially, some genes annotated as *cxcl12* and *cxcr4* were identified from the comparative transcriptomes between the diseased and control individuals (32), suggesting the antiviral role in response to *Ca*HV infection. In this study, we first analyzed the diversification, evolution and biased expression pattern of *cxcr4*/*cxcl12* genes in hexaploid gibel carp. Then, we explored the role of Cxcr4/Cxcl12 axis in the antiviral immune response by blocking CXCR4 with AMD3100 and revealed the related signaling pathways.

## Materials and Methods

### *Ca*HV Infection and Sample Collection

Six-month-old individuals of gibel carp clone F with 67.68 (±2.16) g average weight were collected from the GuanQiao Experimental Station, Institute of Hydrobiology, Chinese Academy of Sciences. Ten tissues (spleen, head kidney, kidney, thymus, gill, intestine, brain, heart, muscle and liver) from three individuals were collected for quantitative reverse transcription PCR (qPCR) analysis before *Ca*HV infection. The *Ca*HV challenge experiments were performed as previously described (32, 39). In brief, sixty individuals were randomly divided into two groups, and were intraperitoneally injected with 500 μl *Ca*HV viral suspension (2.915 × 10^8^ virus particles) or PBS per fish in the experimental group or in control group respectively. Head kidney and spleen were collected from three control (0 day) and infected individuals at 1, 3 and 5 days post injection (dpi), respectively. All samples were preserved in RNAlater (Qiagen, Dusseldorf, Germany) and stored at −20°C for nucleic acid extraction. All experiments were performed in triplicate and the results were representative of three independent experiments.

Healthy individuals were gradually acclimatized with aerated water at 24 (±1) °C for 2 weeks before infection, and fed twice a day. After deep and overdosed anesthesia with styrylpyridine (30–50 mg/L; Aladdin, Shanghai, China), the fish were euthanized by immediately cutting off the spinal cord adjacent to the head. All procedures in this study were approved by the Institutional Animal Care and Use Committee of Institute of Hydrobiology, Chinese Academy of Sciences (protocol number 2016-018).

### Sequence and Phylogenetic Analyses

The complete cDNA sequences of gibel carp *cxcr4*s and *cxcl12*s (GenBank accession numbers MT330400, MT330401, MT330402, MT330403, MT330404, MT330405, MT330406, and MT330407) were amplified by 3′ and 5′ RACE using SMARTer® RACE 5′/3′ Kit (Clontech, San Francisco, USA) from head kidneys of diseased fish after *Ca*HV infection. Amino acid sequences and transmembrane-domain were predicted by ORF Finder (https://www.ncbi.nlm.nih.gov/orffinder/) and SMART (http://smart.embl-heidelberg.de/), respectively. To analyze the evolution of vertebrate Cxcr4 and Cxcl12, the sequence information of 7 species were obtained from the Ensembl Genome browser (http://asia.ensembl.org/index.html) and National Center for Biotechnology Information (NCBI) (https://www.ncbi.nlm.nih.gov/). Multiple protein sequences were aligned by Clustal W program and the phylogenetic tree was constructed by bootstrap analysis (1000 replicates) using the neighbor-joining method (NJ) in MEGA 7.0 software. The exon-intron structure prediction was made by mRNA and genomic sequences alignment of *cxcr4*s and *cxcl12*s. The conserved synteny was analyzed among the chromosomal regions around *cxcr4*s and *cxcl12*s genes in human (*Homo sapiens*), chicken (*Gallus gallus*), spotted gar (*Lepisosteus oculatus*), zebrafish, crucian carp and gibel carp.

### AMD3100 Treatment *in vivo*

One hundred twenty gibel carp individuals were randomly divided into four groups. The healthy and *Ca*HV-infected individuals intraperitoneally injected with 200 μl PBS were considered as negative and positive control group (NC and PC), respectively. In the two experimental groups (E1 and E2), the healthy and *Ca*HV-infected individuals were intraperitoneally injected with CXCR4 antagonist AMD3100 (MedChemExpress, New Jersey, USA) (100 μg in 200 μl PBS). Briefly, PBS or AMD3100 was injected per day until the fish died, and *Ca*HV was injected only once at the 24 h after the first injection of PBS or AMD3100 in PC and E2 group, while NC and E1 group injected PBS instead. The procedure of *Ca*HV infection was described above. After *Ca*HV challenge, the individuals in four groups were monitored every 24 h to score mortality. Head kidney and spleen were collected from individuals at 3dpi to extract RNA for subsequent qPCR assays. The experiments were performed in triplicate.

### RNA Extraction and qPCR

RNA extraction and qPCR were performed as previously described (37, 38). Total RNAs from tissues were extracted using SV Total RNA isolation System (Promega, Madison, USA) according to the manufacturer's protocol. The first-strand cDNAs were synthesized in a 20 μL reaction volume following the protocol of GoScript™ Reverse Transcription System (Promega, Madison, USA). qPCR was performed on a CFX96TM Real-Time PCR System (Bio-Rad, California, USA) using an iTaq™ Universal SYBR® Green Supermix (Bio-Rad, California, USA). Primers used for qPCR analysis were designed with Oligo Calc (Oligonucleotide Properties Calculator) (http://biotools.nubic.northwestern.edu/OligoCalc.html) and listed in Supplementary Table 1. The specificity of each pair of primers was confirmed by sequencing. All samples were analyzed in triplicate, and relative expression levels of target genes were calculated using the 2^−ΔΔCT^ method. The optimal reference gene, *eukaryotic translation elongation factor 1 alpha 1, like 1* (*eef1a1l1*) (M value = 0.74 <1.5) was selected as the normalizer for qPCR (37).

### AMD3100 Treatment *in vitro*

Crucian carp (*C. auratus*) blastula embryonic (CAB) cells were used to perform the Cxcr4 inhibition experiments with a final AMD3100 concentration of 10 μM. Controls were treated with same volumes of ethanol. Cells after 24 h treatment were harvested to extract RNA for subsequent qPCR assays. The experiments were also performed in triplicate and the results were representative of three independent experiments.

### Plasmid Constructs

For luciferase assays, expression plasmids were generated by inserting the full length Open-reading frame (ORF) of *Cgcxcr4*s and *Cgcxcl12*s into the pCS2+ vector (Invitrogen, Carlsbad, USA). The dominant negative mutant plasmids, gibel carp MAPK1-DN (aa 34–369) and MAPK3-DN (aa 70–396) were generated by cloning the corresponding DNA fragments into pcDNA3.1+ vector. Other plasmids, including *Ca*IFNpro-Luc and *Cg*VIPpro-Luc, and some dominant negative mutant plasmids including STAT1a-ΔC and STAT1b-ΔC were constructed previously (40–42). All constructs were verified by sequencing.

### Transfection and Luciferase Activity Assays

CAB cells were seeded overnight in 6-well plates and transfected with *Cg*Cxcr4s-pCS2+ and *Cg*Cxcl12s-pCS2+ respectively using FuGENE HD Transfection Reagent (Promega, Madison, USA) (40, 43). After 24 h transfection, 1 μg/ml polyinosinic:polycytidylic acid (poly(I:C)), was transfected to induce the expression of immune genes (44, 45). The cells were collected in TRIzol reagent (Invitrogen, Carlsbad, USA) after 24 h stimulation. For luciferase activity assay, CAB cells were seeded overnight in 24-well plates and transfected with various constructs at a ratio of 10:10:1 (250 ng *Ca*IFNpro-Luc or *Cg*VIPpro-Luc plasmid: 250 ng corresponding expression plasmid: 25 ng Renilla luciferase plasmid pRL-TK) (40, 43, 44). For the control, the expression plasmid was replaced by empty vector pCS2+. At 24 h post-transfection, the cells were treated again with poly(I:C) infection and the controls were added serum-free medium instead. After 48 h transfection, the cells were harvested and lysed according to the Dual-Luciferase Reporter Assay System (Promega, Madison, USA). Luciferase activities were measured by a Junior LB9509 luminometer (Berthold, Pforzheim, Germany) and normalized to the amounts of Renilla luciferase activities. All experiments were performed in triplicate and the results were representative of three independent experiments.

### Western Blotting Assay

CAB cells were seeded in 6-well plates overnight and treated with ethanol (as a control) and AMD3100 for 24 h. Then, the cells were transfected with 1 μg/ml poly(I:C) or added equal volume serum free medium. After 24 h stimulation, the cell were collected and boiled together with SDS-PAGE protein loading buffer (Beyotime, Wuhan, China) for 8 min, and then analyzed by western blotting using crucian carp IRF3 (*Ca*IRF3) polyclonal Ab (40), zebrafish STAT1 polyclonal Ab (HuaAn, Hangzhou, China) and zebrafish IRF7 monoclonal Ab (HuaAn, Hangzhou, China). The anti-tubulin was purchased from ABclone. To reveal the pathways, CAB cells were seeded in 6-well plates overnight and transfected with different *Cg*Cxcl12s and *Cg*Cxcr4s combinations. After 24 h transfection, the cells were collected as above described, and analyzed by western blotting using p-JAK2 monoclonal Ab (Cell Signaling Technology, Danvers, USA) and p-MAPK monoclonal Ab (Cell Signaling Technology, Danvers, USA).

### Statistical Analysis

qPCR and luciferase activity assay data were shown as means ± SD of three independent experiments, and each performed in triplicate. The statistical analysis was performed by SPSS software (SPSS Inc.) using one-way ANOVA. A probability (*p*) < 0.05 was considered statistically significant (^*^), and *p* < 0.01 was considered extremely significant (^**^).

## Results

### Molecular Characterization and Evolution of Duplicated *cxcr4*/*cxcl12* Genes

Four *cxcr4* genes were identified in gibel carp (Supplementary Figure 1). Based on the identities, we supposed that gibel carp has two groups of paralogues (*cxcr4a* and *cxcr4b*) (caused by the Ts3R), and each of them contains two homoeologues (caused by the allopolyploidy), so they were named as *Cgcxcr4a-A, Cgcxcr4a-B, Cgcxcr4b-A* and *Cgcxcr4b-B* respectively. Different from three alleles (≥99.00% identity) of *Cgbmp15*s or *Cgnanos*2s (46, 47), only one sequence was identified for each homoeologue, which indicates that the alleles of each *Cgcxcr4* gene may be identical. The average identity between homoeologues was 89.65 ± 0.75%, while the average identity between paralogs was about 71.5 ± 0.2% (Supplementary Table 2). The SMART and TMHMM analyses revealed that all *Cg*Cxcr4s possessed 7 hydrophobic transmembrane (TM) domains, four extracellular loops (ECL), four intracellular loops (ICL), an extracellular N-terminus and an intracellular C-terminus (Supplementary Figure 3). The major differences existed in the N-terminus and the ECL3 (Supplementary Figure 3). There were also four *cxcr4* genes in tetraploid crucian carp (2n = 4x = 100 chromosomes), and their identities to the corresponding genes of gibel carp ranged from 97.5 to 100% (Supplementary Table 2). Same as *cxcr4s*, four *cxcl12* genes were also identified from gibel carp and crucian carp, respectively (Supplementary Figures 2, 3).

**Figure 1:**
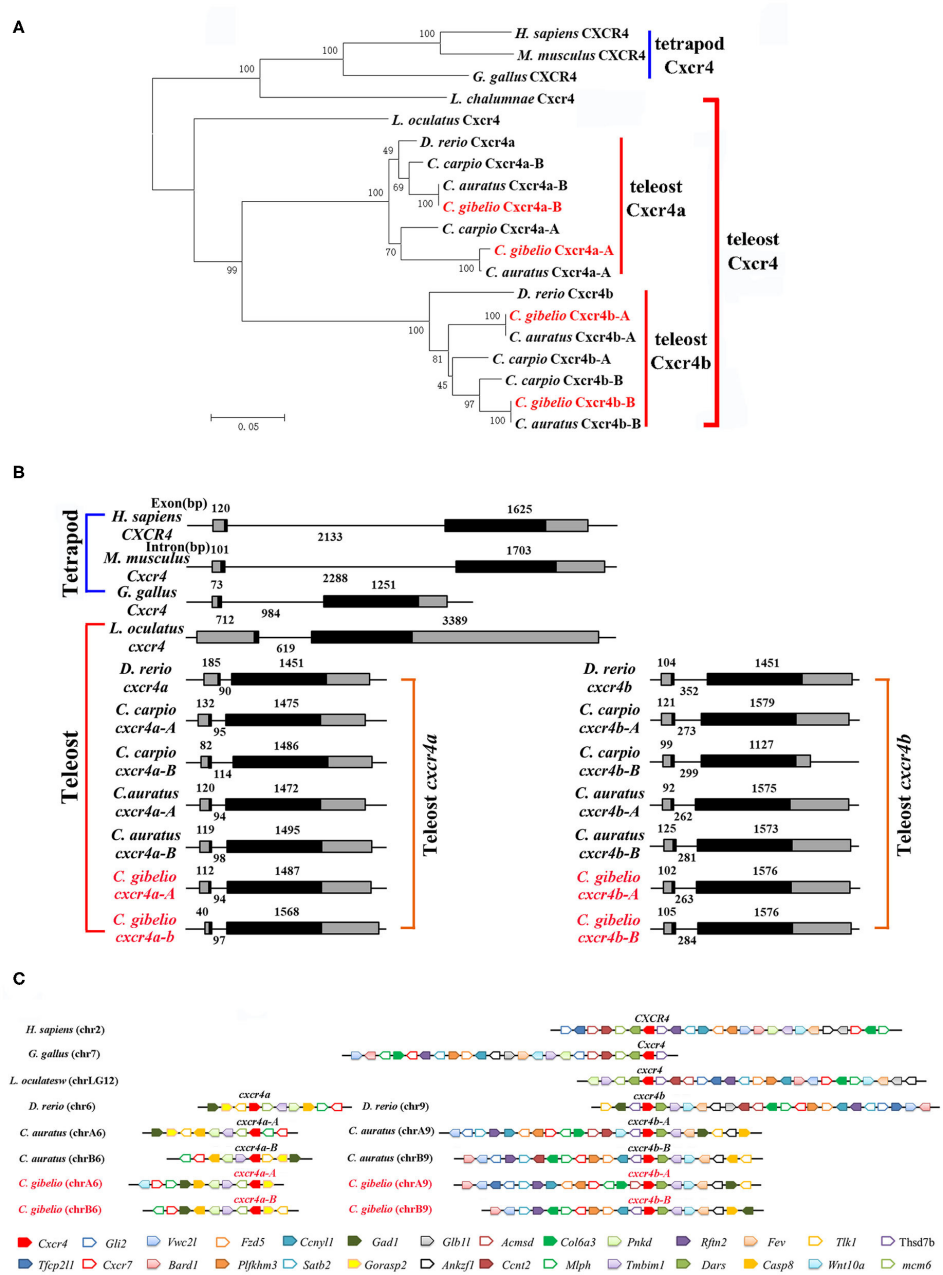
Evolutionary analyses of multiple divergent *cxcr4* genes. **(A)** Phylogenetic tree of Cxcr4 proteins. **(B)** Genomic structures of *cxcr4* genes. Exons and introns are shown by boxes and horizontal lines, respectively. ORFs are highlighted by black boxes. Exon and intron size are indicated above or below as bp. **(C)** Syntenic alignment of chromosomal regions around *cxcr4* genes. Chromosome segments are represented as thick lines. Conserved gene blocks are shown in matching colors and transcription orientations are indicated by arrows. Chr, chromosome.

**Figure 2:**
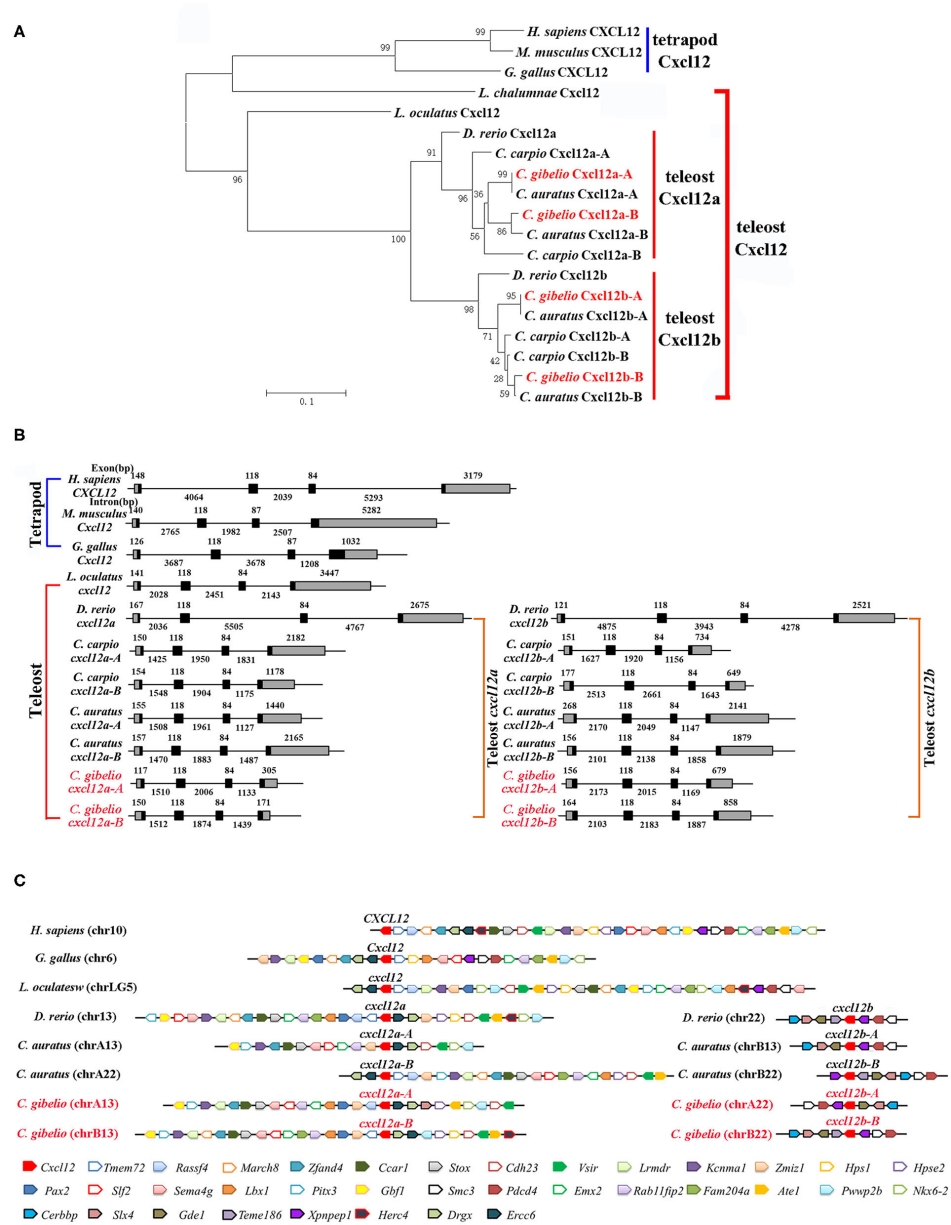
Evolutionary analyses of multiple divergent *cxcl12* genes. **(A)** Phylogenetic tree of Cxcl12 proteins. **(B)** Genomic structures of *cxcl12* genes. Exons and introns are shown by boxes and horizontal lines, respectively. ORFs are highlighted by black boxes. Exon and intron size are indicated above or below as bp. **(C)** Syntenic alignment of chromosomal regions around *cxcl12* genes. Chromosome segments are represented as thick lines. Conserved gene blocks are shown in matching colors and transcription orientations are indicated by arrows. Chr, chromosome.

Consistent with the accepted species phylogeny, *Cg*Cxcr4as and *Cg*Cxcr4bs were first clustered with *Ca*Cxcr4as and *Ca*Cxcr4bs respectively, then clustered with common carp (*Cyprinus carpio*) and zebrafish Cxcr4a and Cxcr4b, respectively (Figure 1A). Teleost Cxcr4a and Cxcr4b branch were grouped together and then clustered with spot gar Cxcr4, while tetrapod CXCR4s and coelacanth Cxcr4 were clustered into another branch (Figure 1A). Similar phylogenetic tree of vertebrate Cxcl12 was shown in Figure 2A. The phylogenies of Cxcr4/Cxcl12 confirmed the assumption that both gibel carp and crucian carp are originated from a common allotetraploid ancestor (27).

We also analyzed the genomic structure and gene synteny of gibel carp *cxcr4s* and *cxcl12s* with other vertebrates. Same to other*s*, gibel carp *cxcr4s* all had a bi-exonic structure (Figure 1B) and *cxcl12s* were all composed of four exons (Figure 2B). *Cgcxcr4a-A, Cgcxcr4a-B, Cgcxcr4b-A*, and *Cgcxcr4b-B* were located on chromosome A6 (chrA6), chrB6, chrA9, and chrB9, respectively. Compared to tetrapod, a complementary loss/retention pattern existed in the vicinity genes of *cxcr4*s between teleost chr6 and chr9 (Figure 1C). Probably owing to the relatively short evolutionary history of allotetraploidy in *Carassius*, the gene loss did not occur between homoeologous chromosomes (chrA6 and chrB6, chrA9 and chrB9) (Figure 1C). Instead, extensive inversions and rearrangements were observed, such as gene blocks *tlk1-gorasp2-gad1, cxcr7-col6a3-mlph*, and *dars-cxcr4-thsd7b* (Figure 1C). The syntenic alignment of *cxcl12* came to the similar conclusions (Figure 2C).

### Dynamic and Differential Expression Patterns of *cxcr4s* and *cxcl12s*

**Figure 3:**
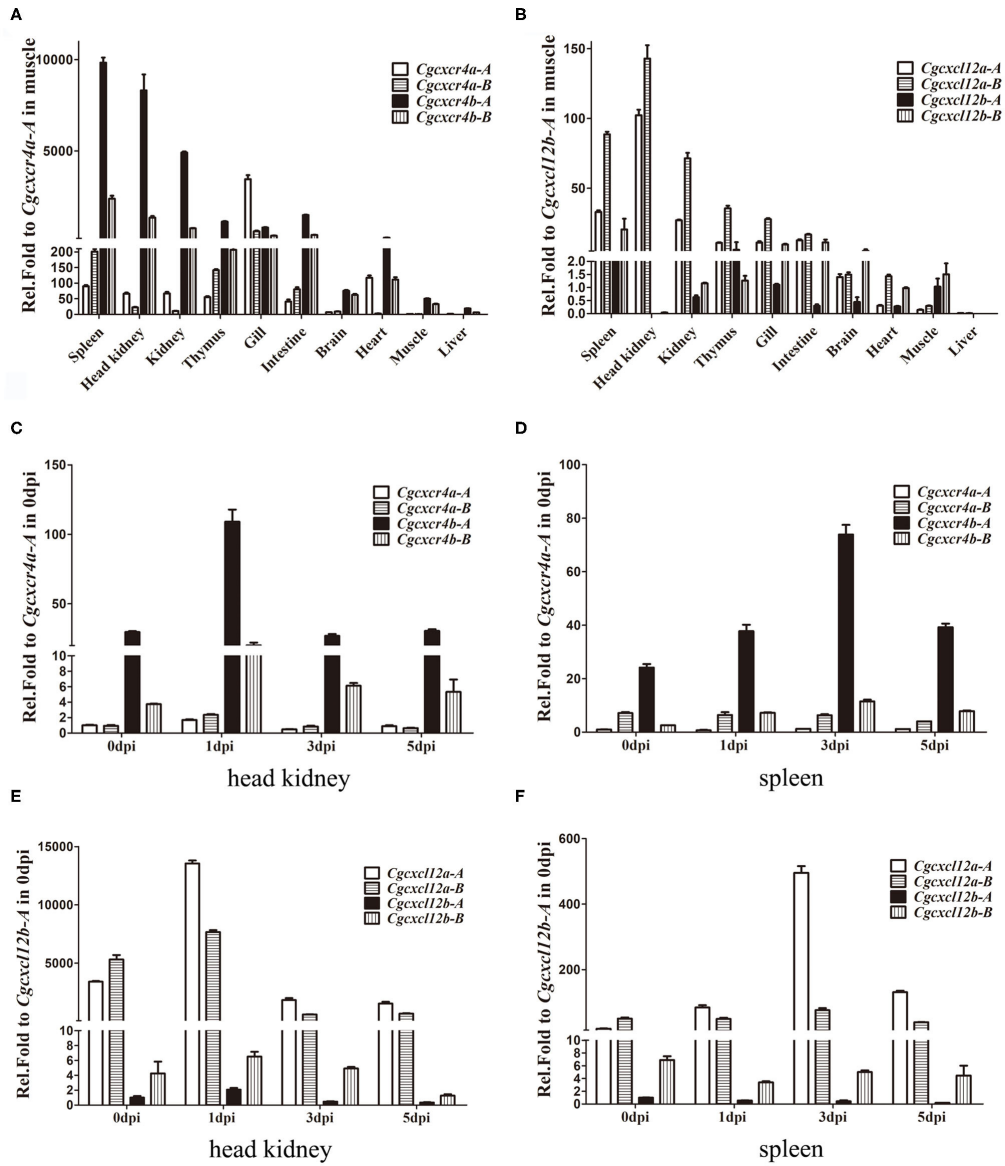
Differential expression patterns of *cxcr4* and *cxcl12* genes. **(A,B)** The expression of *cxcr4* **(A)** and *cxcl12* **(B)** in adult tissues. **(C–F)** The dynamic expression of *cxcr4*/*cxcl12* in head kidney and spleen after *Ca*HV infection, *cxcr4* in head kidney **(C)**, in spleen **(D)**, and *cxcl12* in head kidney **(E)**, in spleen **(F)**. *eef1a1l1* was used as the reference to normalize the templates. Each bar represents the mean ± SEM (*n* = 3).

*Cgcxcr4*s were abundantly expressed in the immune-related tissues, such as spleen, kidney, head kidney and thymus (Figure 3A). Except gill, *Cgcxcr4b*s showed remarkably higher expression than *Cgcxcr4a*s in the analyzed tissues, suggesting that *Cgcxcr4b*s may play a vital role in immune regulation. *Cxcr4* homoeologues also exhibited divergent expression patterns. *Cgcxcr4b-A* was expressed significantly higher than *Cgcxcr4b-B* in most analyzed tissues, while *Cgcxcr4a-A* and *Cgcxcr4a-B* showed well-matched expression bias in different tissues. More abundant *Cgcxcr4a-A* transcripts than *Cgcxcr4a-B* were detected in head kidney, kidney, gill and heart, while reversely differential expression patterns were observed in spleen, thymus, and intestine (Figure 3A). Consistent with the specific chemotactic interaction of grouper Cxcr4b-Cxcl12a (48), *Cgcxcl12a* was the mainly expressed paralog in most analyzed immune-related tissues, as well as *Cgcxcr4b* (Figure 3B). Except in intestine and brain, *Cgcxcl12a-B* showed significantly higher expression than *Cgcxcl12a-A* (Figure 3B). These results indicate that the duplicated *cxcl12* genes also exhibit a differential expression pattern in gibel carp.

Dynamic expression changes of *Cgcxcr4*s or *Cgcxcl12*s in gibel carp after *Ca*HV infection were also evaluated by qPCR (Figures 3C–E). At 1 dpi in head kidney, *Cgcxcr4b-A* and *Cgcxcr4b-B* increased up to 3.7 and 5.4 folds respectively, and then both returned to the basic expression level (Figure 3C). Although fewer *Cgcxcr4a* transcripts were detected in head kidney compared to those of *Cgcxcr4b*, they were also slightly up-regulated (1.7–2.5 folds) at 1 dpi (Figure 3C). In spleen, *Cgcxcr4b-A* and *Cgcxcr4b-B* increased their expression and reached a peak at 3 dpi (Figure 3D). No significant expression change of *Cgcxcr4a*s was detected in spleen after *Ca*HV challenge (Figure 3D). For four *Cgcxcl12* genes, only *Cgcxcl12a-A* was significantly up-regulated in head kidney (4.0 folds at 1 dpi) and spleen (25.5 folds at 3 dpi) after *Ca*HV infection (Figures 3E,F).

### *Cg*Cxcr4/*Cg*Cxcl12 Axis Controls Antiviral Response Against *Ca*HV

**Figure 4:**
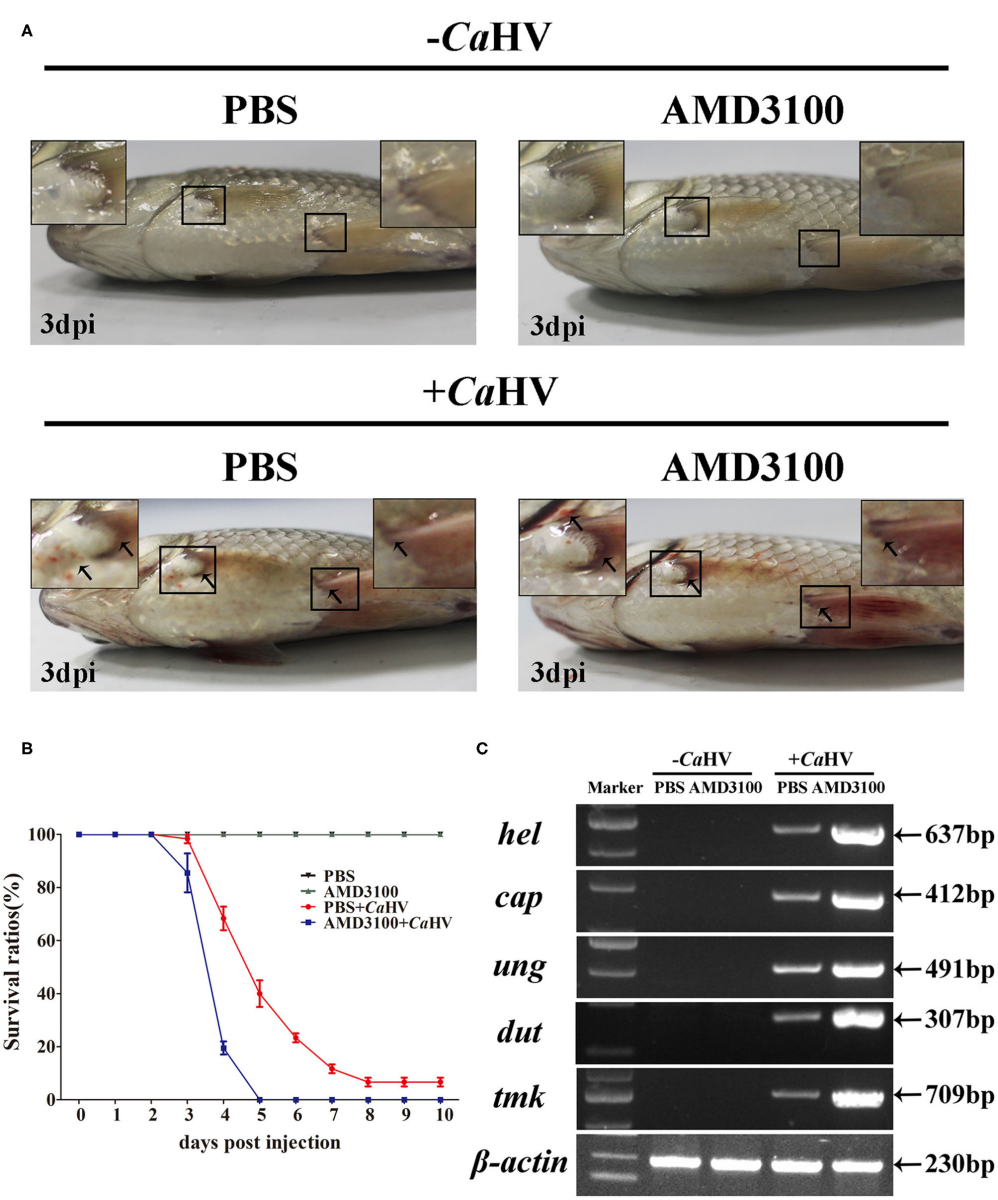
Gibel carp treated with AMD3100 were more sensitive to *Ca*HV infection. **(A)** Representative images of gibel carp, both un-infected group (–*Ca*HV) and infected group (+*Ca*HV) were photographed at third day after intraperitoneal injection PBS (–*Ca*HV) or *Ca*HV (+*Ca*HV), along with the treatment of vehicle (PBS; the control) or AMD3100 (100 μg in 200 μl PBS) every 24 h. Un-infected gibel individuals appeared normal while infected individuals exhibited hemorrhage at the base of fins and on abdomen (black arrows), and AMD3100 treated gibel carp had more severe bleeding symptom. **(B)** Survival ratios after *Ca*HV infection. We counted the numbers of dead fish every 24 h post-infection. The values are the mean ± SEM from three replicate tanks. **(C)** Electrophoretogram of the PCR amplified products of five *Ca*HV genes (*hel, cap, ung, dut*, and *tmk*) in four groups (NC, E1, PC, and E2). Gene symbols and the sizes of amplified products are indicated by the left and right side of the figure, respectively.

To explore the role of *Cg*Cxcr4/*Cg*Cxcl12 axis in antiviral response to *Ca*HV, we investigated the symptoms and the *Ca*HV abundance among four gibel carp groups treated with/without AMD3100 that specifically blocks the binding between CXCR4 and CXCL12 (49, 50). AMD3100 injection did not affect the survival of the healthy individuals (not challenged by *Ca*HV) (Figures 4A,B). After *Ca*HV infection, the individuals exhibited hemorrhage at the base of fins and on abdomen at 3 dip (arrows in Figure 4A). Cxcr4-blocked individuals showed more severe bleeding and lower survival rate (Figures 4A,B), indicating more susceptibility to *Ca*HV infection than control individuals (injecting PBS). The death started at 3 dpi in Cxcr4-blocked group and all died at 5 dpi, while the first death of control group occurred at 4 dpi and about 7% survivors appeared healthy at 10 dpi (Figure 4B). To further confirm this susceptibility induced by Cxcr4 inhibition, we detected the abundances of five *Ca*HV genes to evaluate the *Ca*HV replication (38). After 35 amplification cycles, no DNA fragment of *Ca*HV was detected from the healthy individuals, while the specific bands were amplified in the *Ca*HV-infected individuals (Figure 4C). Accordant with more severe symptoms, the Cxcr4-blocked fish had markedly stronger bands than the control individuals at 3 dpi (Figure 4C).

**Figure 5:**
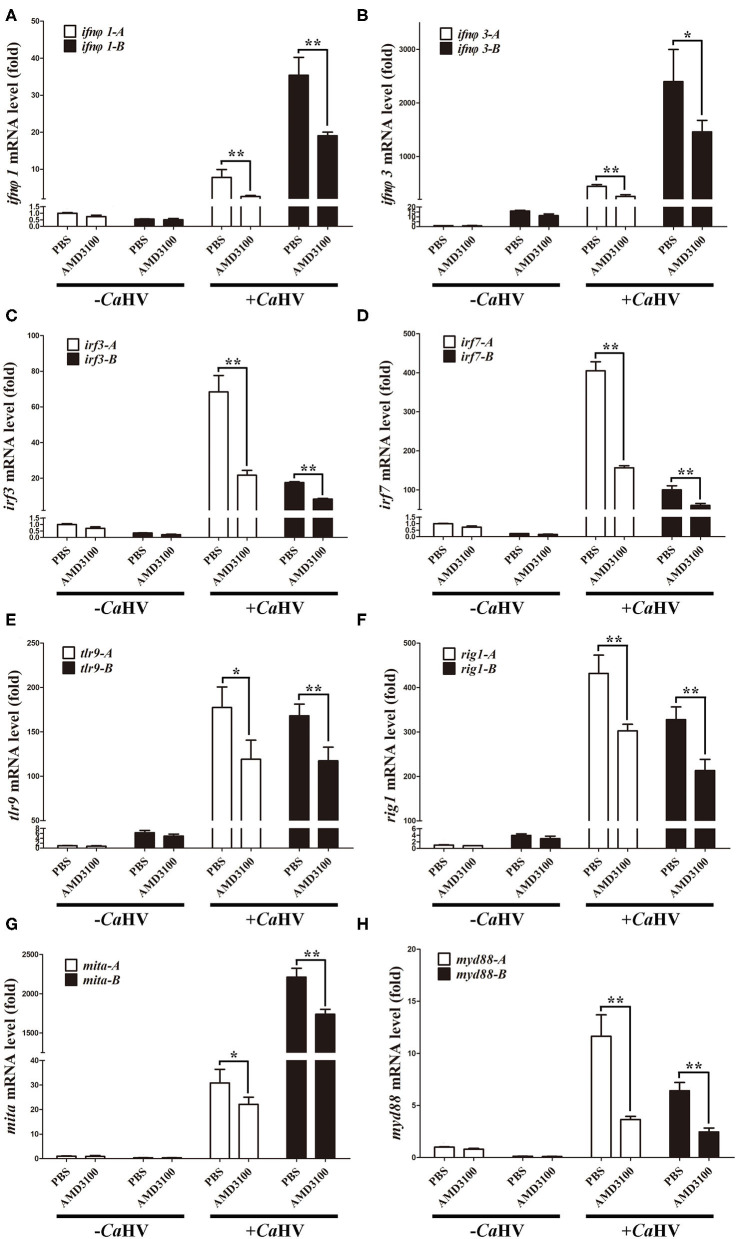
AMD3100 suppressed *Ca*HV-induced activation of key antiviral genes in gibel carp head kidney. **(A–H)** qPCR analysis of *ifn*φ*1* **(A)**, *ifn*φ*3* **(B)**, *irf3* **(C)**, *irf7* **(D)**, *tlr9* **(E)**, *rig1* **(F)**, *mita* **(G)**, and *myd88* **(H)** in AMD3100 treated gibel carp after *Ca*HV infextion. *eef1a1l1* was used as control. Each bar represents mean ± SEM (*n* = 3). The asterisks indicate the significant differences (*p < 0.05, **p < 0.01).

We also analyzed the expression of several key antiviral genes in head kidney and spleen (37), such as *interferon* (*ifn*φ*1* and *ifn*φ*3*), *interferon regulatory factor* (*irf3* and *irf7*), host pattern recognition receptors PRRs [*toll-like receptor 9* (*tlr9*) and *retinoic acid induce gene I* (*rig1*)] and PRR-mediated IFN signal pathway genes [*mediator of irf3 activation* (*mita*) and *myeloid differentiation primary-response gene 88* (*myd88*)]. Similar to *cxcr4* and *cxcl12*, they all possessed A and B homoeologues with divergent sequences and with biased expression in gibel carp (Figure 5). After *Ca*HV infection, they were all up-regulated, and the up-regulation folds were significantly suppressed when Cxcr4-Cxcl12 binding was blocked (Figure 5 and Supplementary Figure 4). Together with above results, we suggest that blocking Cxcr4/Cxcl12 axis should suppress gibel carp antiviral response to *Ca*HV.

### *Cg*Cxcl12/*Cg*Cxcr4 Axis Enhances Cellular Antiviral Response Through MAPK3 and JAK/STAT Pathway

**Figure 6:**
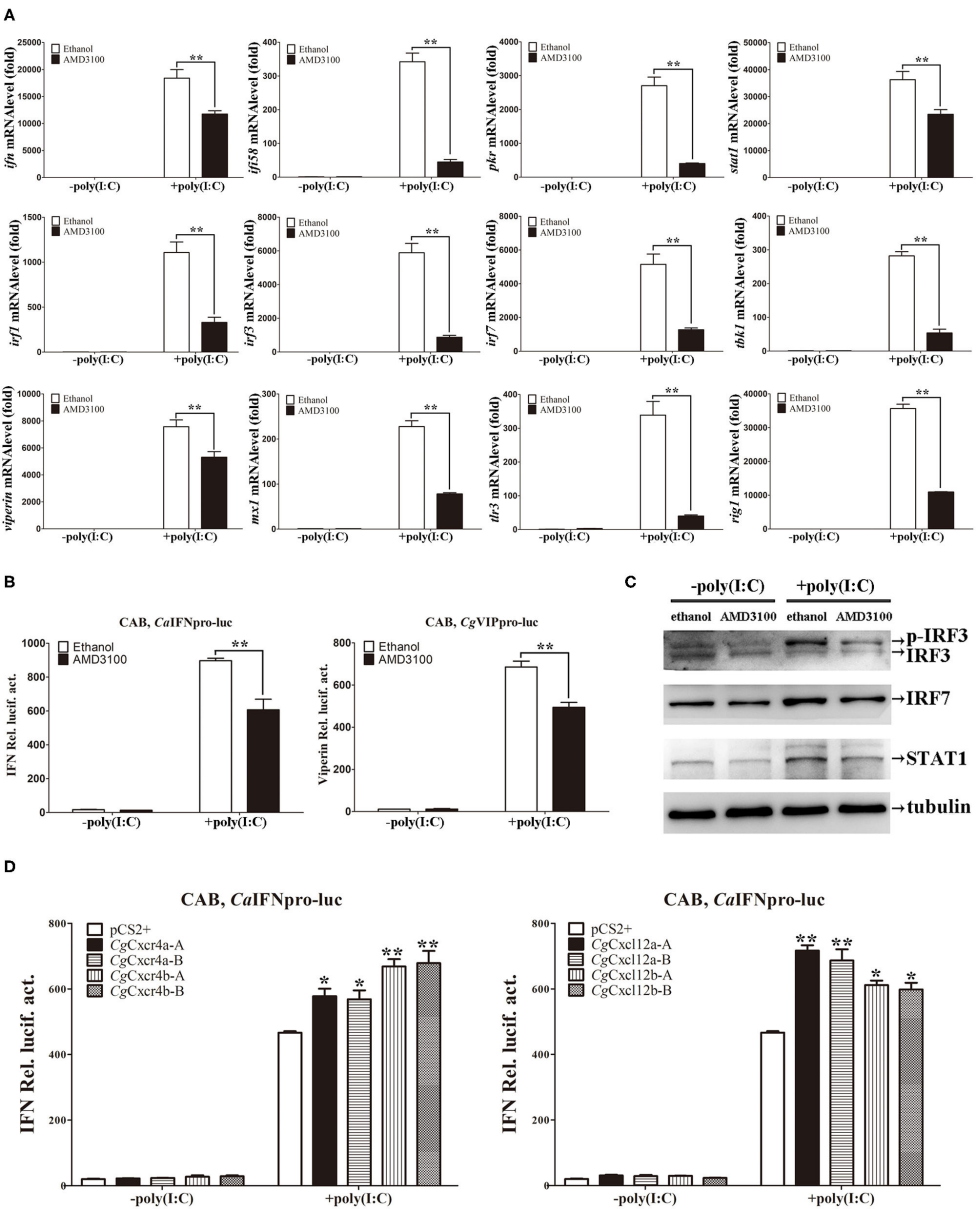
*Cg*Cxcl12s and *Cg*Cxcr4s facilitated cellular antiviral response. **(A)** Treatment of CAB cells with 1μM AMD3100 after poly(I:C) stimulation suppressed the up-regulation expression of *ifn, ifi58, pkr, stat1, irf1, irf3, irf7, tbk1, viperin, mx1, tlr3*, and *rig1*. **(B)** AMD3100 inhibited the activation of fish IFN and *viperin* promoters in CAB cells after poly(I:C) stimulation. **(C)** The up-regulation protein expression of p-IRF3, IRF7, and STAT1 were suppressed in AMD3100-treated CAB cells after poly(I:C) stimulation. **(D)** Poly(I:C)-triggered activation of IFN promoter was enhanced by overexpression of Cxcr4s or Cxcl12s. Each bar represents mean ± SEM (*n* = 3). The asterisks indicate the significant differences (*p < 0.05, **p < 0.01).

The function and signal pathways of about 20 IFN system genes have been well documented in CAB cells (40, 41, 51–53), so we used CAB cells to explore the antiviral response pathways related to *Cg*Cxcr4/*Cg*Cxcl12 axis. After treatment with poly(I:C) that mimics the double-strand RNA (dsRNA) virus infection, all of the analyzed IFN system genes, including *ifn, ifi58* (*interferon inducible protein 58*), *pkr* (*dsRNA-activated protein kinase R*), *irf1* (*interferon regulatory factor 1*), *irf3, irf7, viperin, mx1* (*MX dynamin-like GTPase 1*), *tbk1* (*TANK binding kinase 1*), *stat1*(*signal transducer and activator of transcription 1*), *tlr3* (*toll-like receptor 3*) and *rig1*, were significantly up-regulated (Figure 6A). Consistent with the results in *vivo* (Figure 5), the similar suppression effects were also observed in the CAB cells treated with AMD3100 (Figure 6A). Furthermore, ADM3100 pre-treatment also inhibited the activities of *Ca*IFN and *Cg*Vip promoters, as well as elevating the amounts of STAT1, IRF7 and phosphorylated IRF3 (40), induced by poly(I:C) stimulation (Figures 6B,C). Finally, both *Cg*Cxcr4 or *Cg*Cxcl12 overexpression (Supplementary Figure 5) promoted the activation of IFN promoter induced by poly(I:C) (Figure 6D). In addition, The up-regulation folds affected by *Cg*Cxcr4bs or *Cg*Cxcl12as were bigger than their paralogues respectively, and no obviously differences observed between each homoeologous pair (*Cg*Cxcr4b-A: 33.7, *Cg*Cxcr4b-B: 34.2, *Cg*Cxcr4a-A: 29.2, *Cg*Cxcr4a-B: 28.7; *Cg*Cxcl12a-A: 36.2, *Cg*Cxcl12a-B: 34.1, *Cg*Cxcl12b-A: 31.3 and *Cg*Cxcl12b-B:30.2) (Figure 6D). Considering the bias expression in immune organs, we suppose that Cxcr4b and Cxcl12a might be the major antiviral couple in gibel carp. Altogether, Cxcr4/Cxcl12 axis facilitates the cellular antiviral response stimulated by poly(I:C).

**Figure 7:**
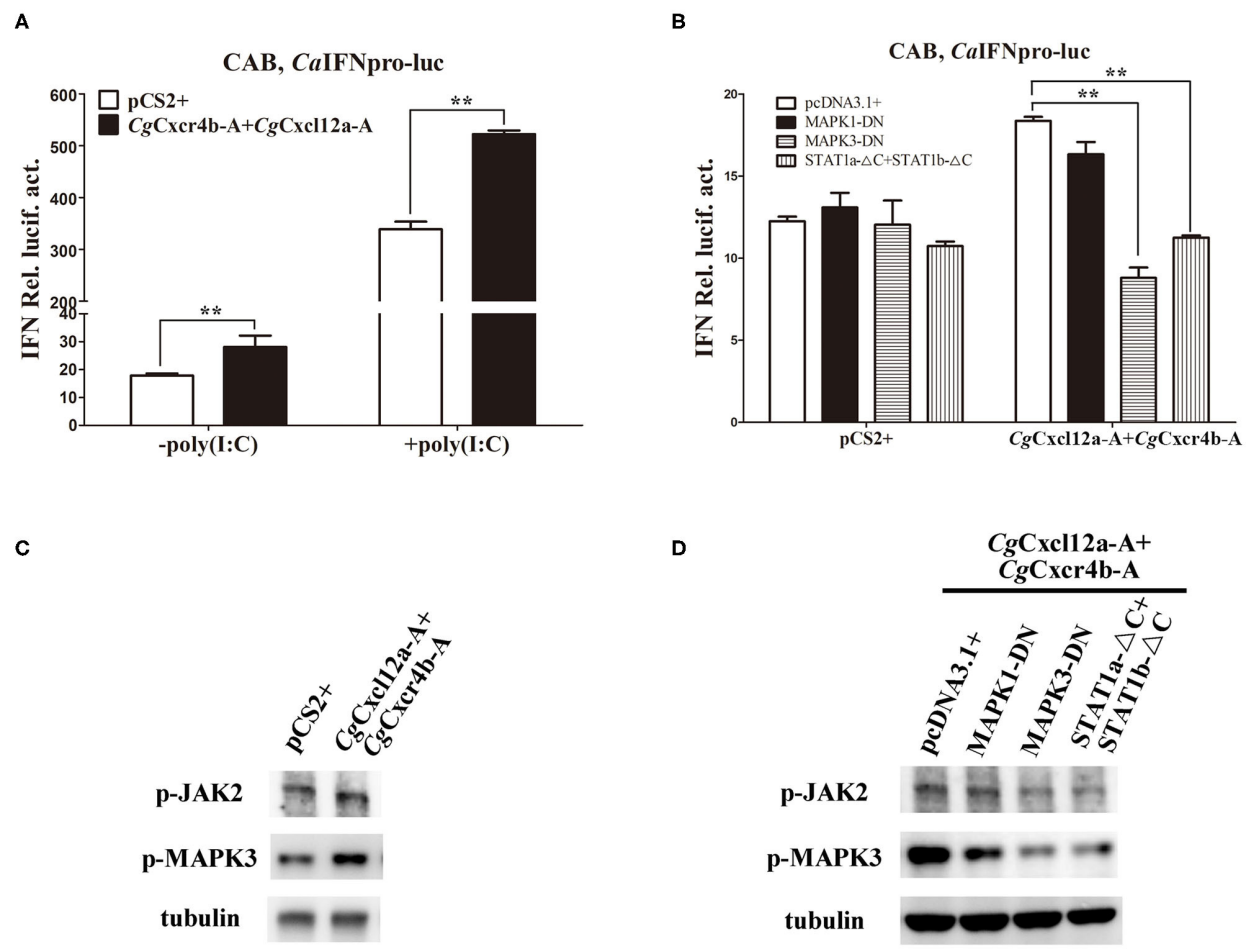
Cxcr4/Cxcl12 axis induced IFN activity via MAPK3 and JAK/STAT pathways. **(A)** *Cg*Cxcl12a-A and *Cg*Cxcr4b-A combination activated the activity of *Ca*IFN promoter. **(B)** The activation of *Ca*IFN promoter induced by *Cg*Cxcl12a-A and *Cg*Cxcr4b-A overexpressing was inhibited by overexpression of dominant negative mutants of MAPK and JAK/STAT signaling molecules. **(C)** Overexpression of *Cg*Cxcl12a-A and *Cg*Cxcr4b-A increased the protein expression of p-JAK2, STAT1, p-MAPK3 and MAPK3. **(D)** The up-regulation protein expression of p-JAK2, STAT1, p-MAPK3, and MAPK3 were suppressed by transfecting dominant negative mutants MAPK3-DN and STAT1a-ΔC+STAT1b-ΔC. Each bar represents mean ± SEM (*n* = 3). The asterisks indicate the significant differences (**p < 0.01).

To avoid the possible bias effects of poly(I:C), we co-transfected different *Cg*Cxcr4s-*Cg*Cxcl12s combinations in CAB cells to directly detect the *Ca*IFN promoter luciferase activities. Compared to the control, co-transfection of *Cg*Cxcl12a-A-*Cg*Cxcr4b-A combination resulted in stronger activation of *Ca*IFN promoter (Figure 7A), and other different ligand-receptor combinations showed the similar results (Supplementary Figure 6). CXCR4/CXCL12 axis can activate PI3K/Akt, PLC/IP3, ERK1/2 pathways (14, 54), and β-arrestin/JAK/STAT pathway in mammals (55, 56). To reveal the molecular mechanisms by which Cxcr4/Cxcl12 axis regulates IFN expression, we co-transfected *Cg*Cxcr4b-A and *Cg*Cxcl12a-A with dominant negative mutants of MAPK1, MAPK3 or STAT1 (MAPK1-DN, MAPK3-DN or STAT1a-ΔC+STAT1b-ΔC) in above *vitro* system. After 24 h of transfection, the activity of *Ca*IFN promoter was diminished by the co-overexpression of MAPK3-DN or STAT1a-ΔC+STAT1b-ΔC, while overexpression of MAPK1-DN did not obviously affect the induction of IFN expression (Figure 7B). To further verify the results, we used western blotting to detect the expression of phosphorylation JAK2 (p-JAK2) and p-MAPK3 proteins. As shown in Figure 7C, overexpression of *Cg*Cxcr4b-A and *Cg*Cxcl12a-A promoted the expression of p-JAK2 and p-MAPK3. The increased expression was significantly reduced by co-transfecting MAPK3-DN or STAT1a-ΔC+STAT1b-ΔC (Figure 7D). These results indicate that *Cg*Cxcl12/*Cg*Cxcr4 axis can regulate IFN expression through MAPK3 and JAK/STAT pathways.

## Discussion

Polyploidy is considered as a major driver of species diversification and frequently occurred during teleost evolution (25, 57, 58). Although the consequences are complex and variable, it might increase short-term adaptive potential and long-term evolvability for polyploid organisms along with subsequent rediploidization (59–61). Ts3R was supposed to result in two paralogs of each gene compared to tetrapods (28, 62–64). Gibel carp retains two *cxcr4* and *cxcl12* paralogs (*cxcr4a* and *cxcr4b, cxcl12a*, and *cxcl12b*) (Figures 1, 2; Supplementary Figures 1–3). In addition, polyploidy occurred repeatedly in Cypriniformes which further increase genetic variation (28, 65, 66). We identified two homoeologues (A and B) of each paralog from common carp, crucian carp and gibel carp (Figures 1, 2; Supplementary Figures 1–3). Similar to salmons that experienced an extra WGD 95 million years ago, diversification is a major evolution force of duplication genes (67).

During rediploidization, the sub-functionalization and neo-functionalization are the major maintenance ways of gene duplicates (68, 69). The dynamic expression pattern was observed in some fishes after pathogen infection, elucidating that *cxcr4*/*cxcl12* is not only important in immune defense against bacterial and viral infection but also may restrain the immune system in some other physiological processes (17–20). In gibel carp, both paralogous and homoeologous pairs of *cxcr4b* and *cxcl12a* show dynamic and differential expression patterns and antiviral responses. Our results indicate that *cxcr4bs* and *cxcl12as* should play a dominant role in immune response and confirm the specific ligand-receptor recognitions of Cxcl12b and Cxcr4a (48, 70). Although derived from the common ancestor (71), the expression patterns of duplicated genes evolved divergently after separation. For example, a common carp *cxcl12b* (*Cccxcl12b*) exhibits highest identity with *Cgcxcl12b-B*, and they are both highly expressed in the brain (72). But besides, *Cgcxcl12b-B* also shows high expression in spleen (Figure 3B). In gibel carp, all *cxcr4* and *cxcl12* paralogues/homoeologues can promote IFN gene expression in CAB cells, although Cxcl12as and Cxcr4bs show a bit higher ability (Figure 6D). It seems that the sub-functionalization and neo-functionalization of *cxcr4s* or *cxcl12s* mainly occur at the transcriptional level, and *cxcr4* or *cxcl12* copies may still overlap for some functions (68, 73).

Although CXCR4 blockade benefits to autoimmune diseases, it is deleterious in acute inflammation and ischemia, and has a negative impact on negative physiological defense (74–76). In mammals, CXCR4 triggered by CXCL12 binding activates anti-inflammatory signaling pathways and inhibits inflammation (77, 78). It increases the production of pro-inflammatory cytokines, including IFN-γ, TNF-α, IL-6 and so on (79, 80). AMD3100 administration also results in the significant reduction of IFN-γ, TNF-α and IL-6 in the acute stage of ischemic stroke or after spinal cord injury (11, 12). In addition, interruption of the CXCR4/CXCL12 axis not only down-regulates the expression of IFN-γ and TNF-α, but also negatively influences TLR4 signaling and leads to the decrease of inflammatory responses in the LPS treatment mice (76, 81). In zebrafish, Cxcl12/Cxcr4 axis plays an important role to antagonize wound-induced inflammatory signals, but Cxcl12/Cxcr4 signaling also leads to the inappropriate retention of neutrophils at the inflammatory sites (8, 23, 82). Interestingly, impairment in the Cxcl12a/Cxcr4b signaling axis does not affect the recruitment of neutrophils when cxcr4b mutants are infected by *Salmonella typhimurium* (8). Therefore, the role of Cxcl12/Cxcr4 signaling axis against infection may be not affected by the retention and/or migration of leukocyte population. In this study, Cxcr4 blockade not only promotes the duplication of *Ca*HV, but also suppresses the up-regulation expression of the virus recognition receptors *tlr9, tlr3* and the following downstream antiviral genes, both in gibel carp after *Ca*HV infection and CAB cells with poly(I:C) stimulation (Figures 4–6). Moreover, overexpression of *Cg*Cxcr4s or *Cg*Cxcl12s both instigate the induction of IFN by poly(I:C) (Figure 6D). The innate immune response triggered by virus infection increases the expression of IFN and downstream IFN-stimulated genes (ISGs) to protect the host both in mammals and teleost (53, 83, 84). Taken together, we suggest that Cxcr4/Cxcl12 axis may be important for the innate immune responses to resist virus invasion in teleost.

Cxcl12 was reported to enhance dimerization of Cxcr4, and then to activate the downstream pathways (85). Activating some signaling pathways such as ERK, JAK/STAT and PI3K can regulate the transcription factors, including AP1, NF-κB and NFAT, to regulate the expression of cytokines (86–89). They also play some roles in IFN induction by virus infection, as their domain negative mutants markedly inhibited the induction of IFN (Figure 7B). Following CXCl12 binding, CXCR4 activates the Janus Kinases by changing their conformation to promote the JAK/STAT signaling pathway and the downstream transcription factor NF-κB that stimulates IFN secretion (85, 90, 91). In addition, CXCR4 internalization also activates the β-arrestin pathway, which severs as a scaffold for ERK and enhance the ERK activation (56, 92). In our study, blocking MAPK3 and STAT1 has a significant suppression effect on IFN induction by *Cg*Cxcr4b-A and *Cg*Cxcl12a-A over-expression (Figure 7), indicating MAPK and JAK/STAT maybe the major pathways that *Cg*Cxcr4s/*Cg*Cxcl12s axis participates in antiviral response. However, blocking MAPK1 does not affect the induction of IFN, and the MAPK1 protein is much less than MAPK3 in CAB cells (data not shown), which suggest that MAPK1 might play a different role from MAPK3 in teleost.

In conclusion, the current study represents the diversification and dominant expression in immune tissues of Cxcl12/Cxcr4 axis in hexaploid gibel carp. It is the first time to report its function in teleost antiviral response, which regulates IFN expression mainly through MAPK3 and JAK/STAT pathways. These findings would expand our knowledge about the complex function of Cxcl12/Cxcr4 axis in teleost, which is a link between the innate and the adaptive immune system and play a key role to improve the adaptive immunity (22, 93). Understanding the regulation mechanisms of Cxcl12/Cxcr4 signaling can provide useful information for disease control with effective immune protection in gibel carp. Further study of Cxcl12/Cxcr4 signaling and its downstream factors will help us to better understand the mechanism of host against *Ca*HV infection, and provide resistant-related candidate genes for disease-resistance breeding of gibel carp.

## Data Availability Statement

The raw data supporting the conclusions of this article will be made available by the authors, without undue reservation, to any qualified researcher.

## Ethics Statement

The animal study was reviewed and approved by Institutional Animal Care and Use Committee of Institute of Hydrobiology, Chinese Academy of Sciences (protocol number 2016-018).

## Author Contributions

W-JL and F-XG performed the experiments, analyzed the data, and drafted the manuscript. YW participated in the data analysis and revised manuscript. ZL participated in the sample collection. Y-LZ and X-JZ participated in the data analysis. LZ designed the studies, analyzed the data, and drafted the manuscript. J-FG conceived the study and revised the manuscript. All authors contributed to the article and approved the submitted version.

## Conflict of Interest

The authors declare that the research was conducted in the absence of any commercial or financial relationships that could be construed as a potential conflict of interest.

## Supplementary Material

Supplementary Figure 1 Nucleotide sequences and deduced amino acid sequences of four *Cgcxcr4* genes.

Supplementary Figure 2 Nucleotide sequences and deduced amino acid sequences of four *Cgcxcl12* genes.

Supplementary Figure 3 Multiple amino acid sequence alignment of Cxcr4/Cxcl12 proteins.

Supplementary Figure 4 AMD3100 suppresses *Ca*HV-induced activation of key antiviral genes in gibel carp spleen.

Supplementary Figure 5 The transfection efficiency and subcellular localization of *Cg*Cxcr4s.

Supplementary Figure 6 Different *Cg*Cxcl12 and *Cg*Cxcr4 combinations activated the activity of *Ca*IFN promoter.

Supplementary Table 1 Primers used in this study.

Supplementary Table 2 The amino acid sequence identities of gibel carp and other vertebrate Cxcr4s/Cxcl12s.
