# Supplementary figures and images for: Dynamic and Differential Expression of Duplicated Cxcr4/Cxcl12 Genes Facilitates Antiviral Response in Hexaploid Gibel Carp
Source: Front Immunol. 2020 Sep 11;11:2176. doi: 10.3389/fimmu.2020.02176 (PMC7516010; doi:10.3389/fimmu.2020.02176)

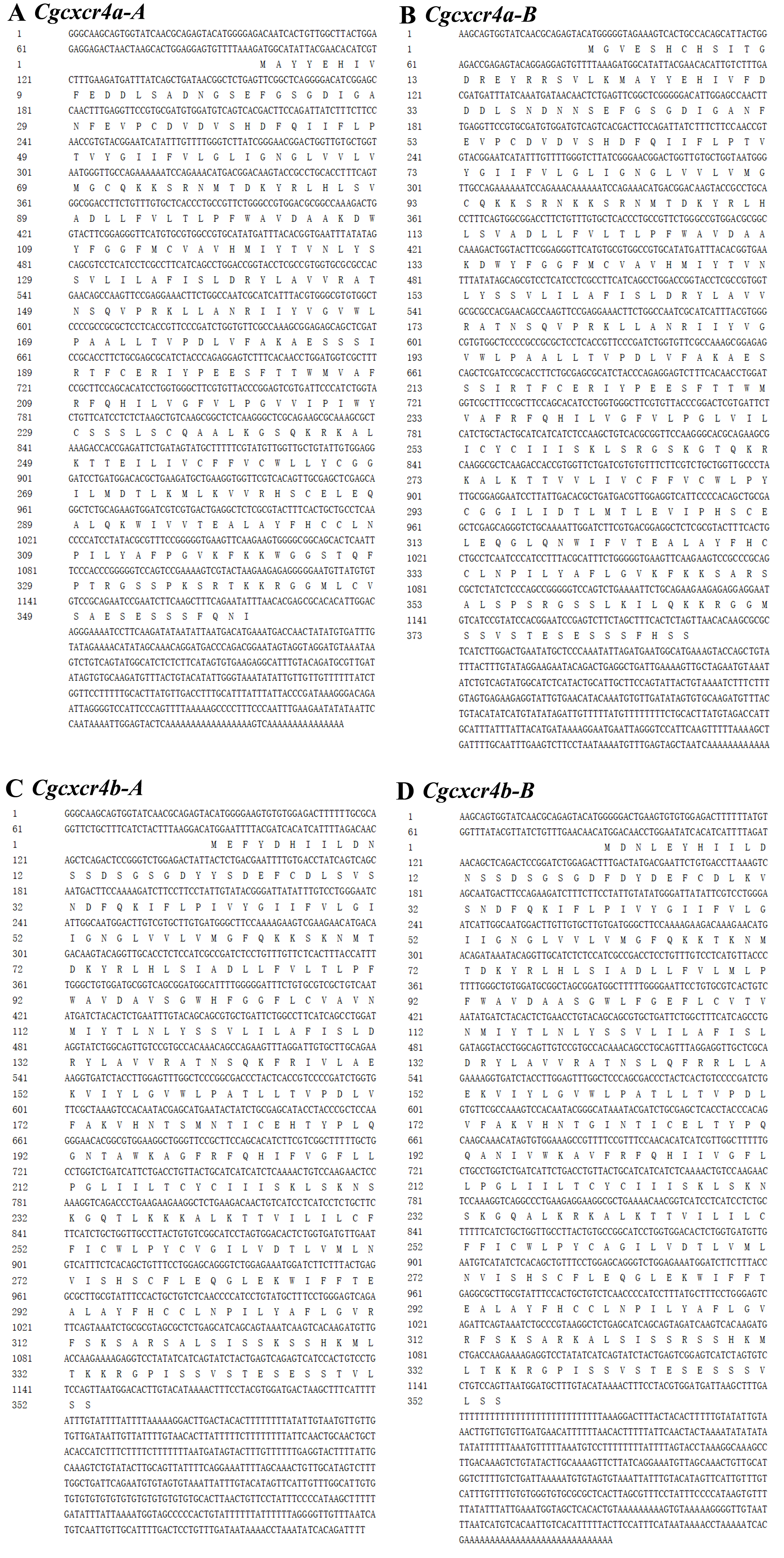

Supplement: Supplementary Figure 1 — Nucleotide sequences and deduced amino acid sequences of four Cgcxcr4 genes. [file Image_1.TIF]

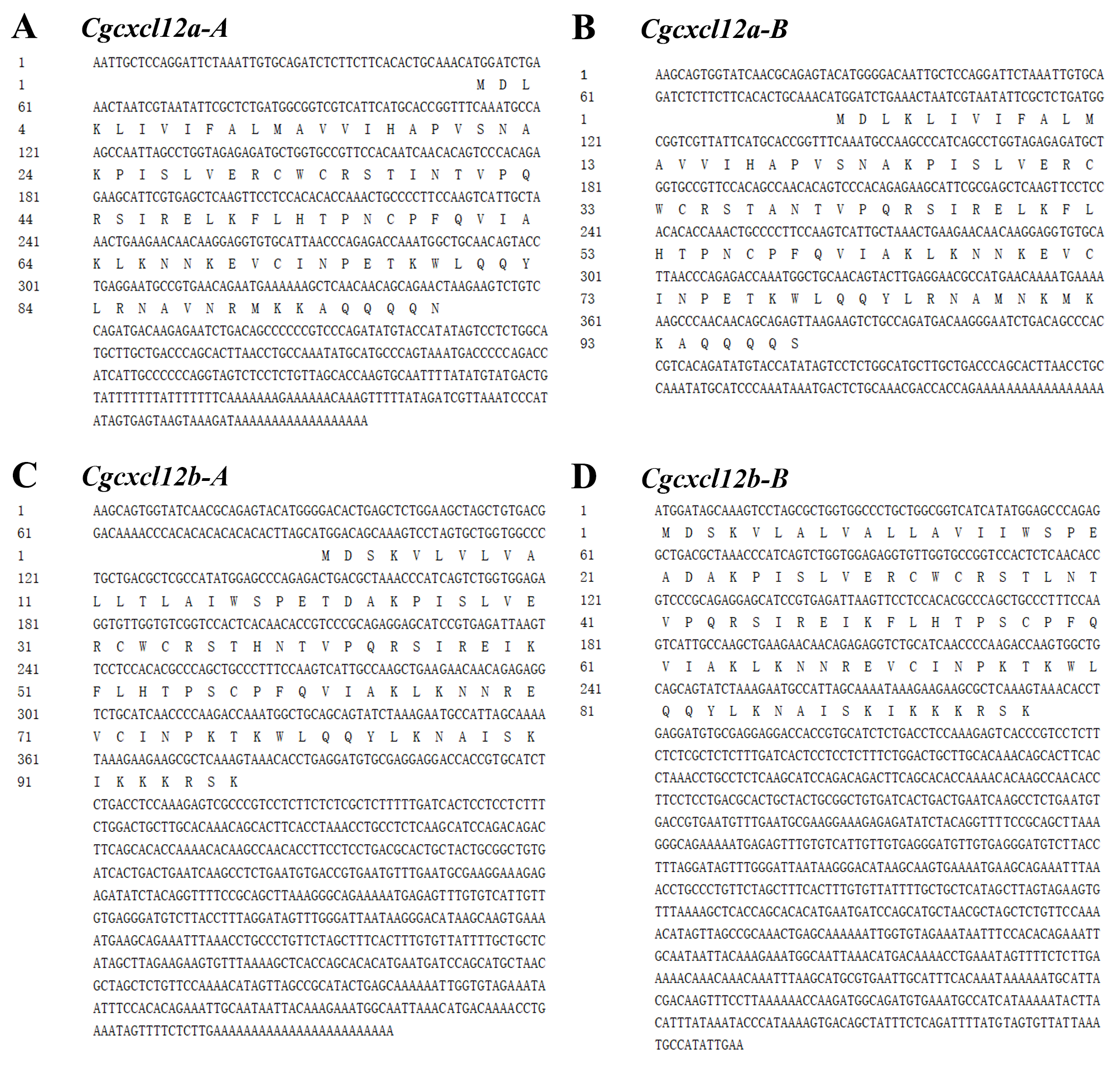

Supplement: Supplementary Figure 2 — Nucleotide sequences and deduced amino acid sequences of four Cgcxcl12 genes. [file Image_2.TIF]

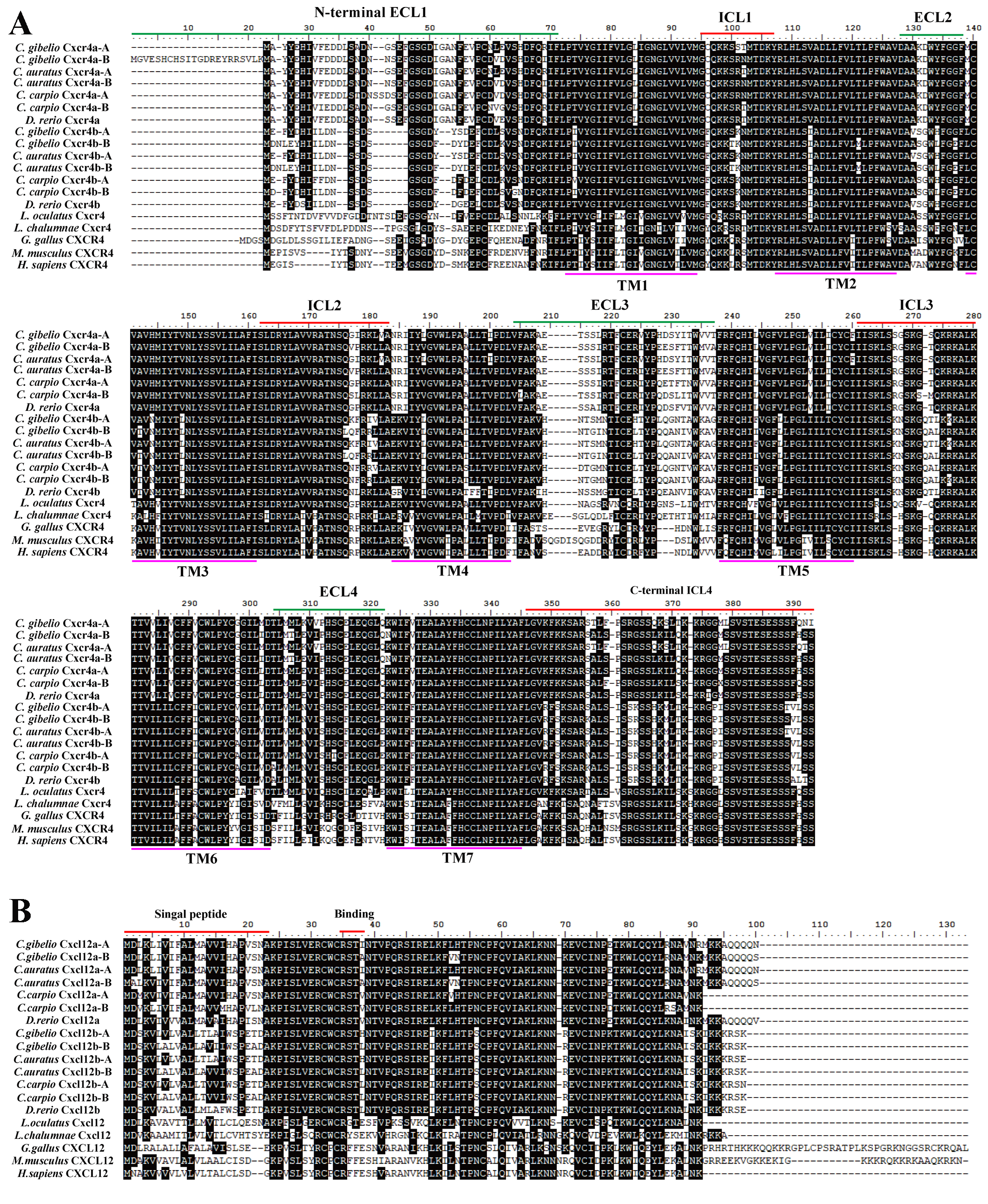

Supplement: Supplementary Figure 3 — Multiple amino acid sequence alignment of Cxcr4/Cxcl12 proteins. [file Image_3.TIF]

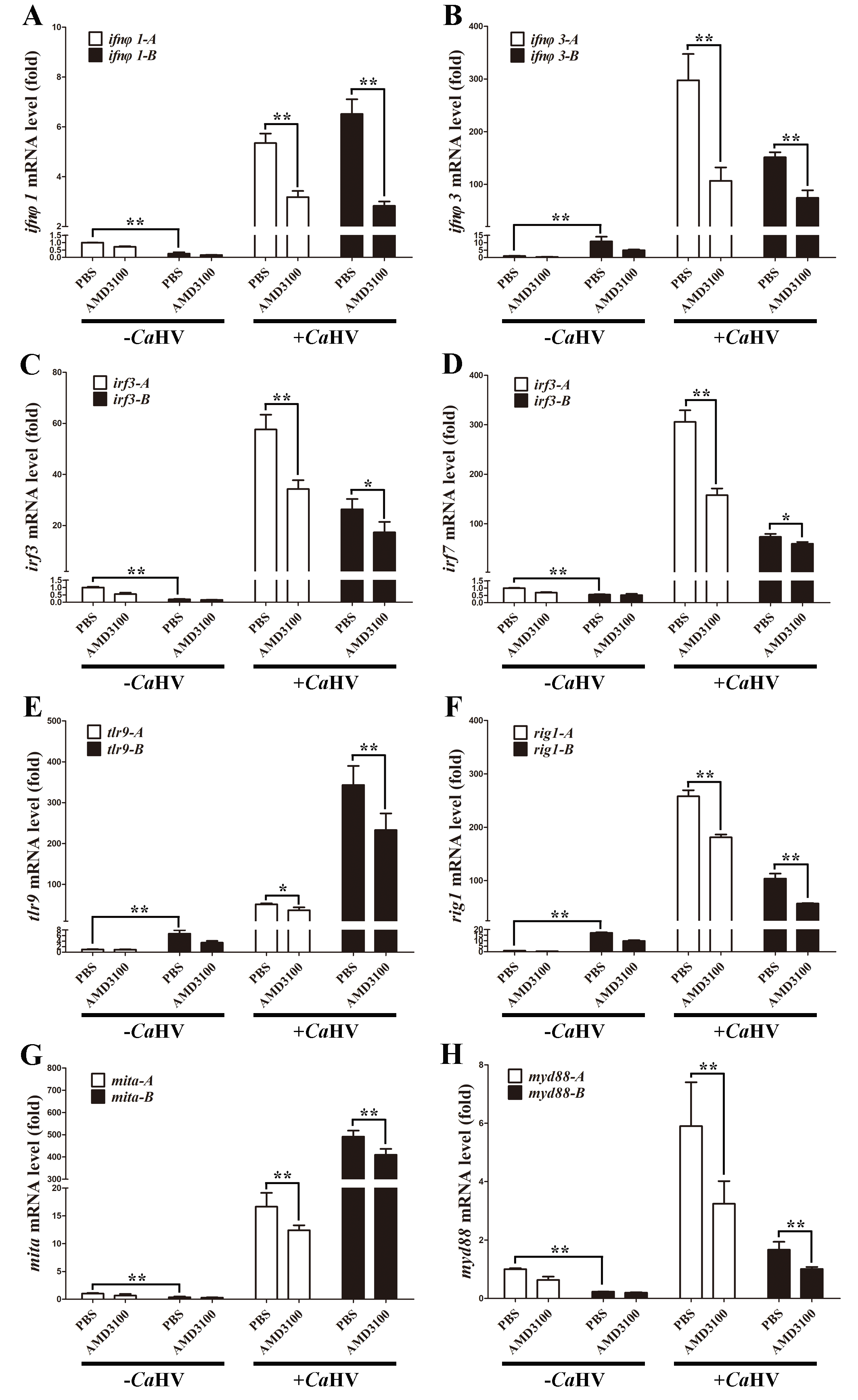

Supplement: Supplementary Figure 4 — AMD3100 suppresses CaHV-induced activation of key antiviral genes in gibel carp spleen. [file Image_4.TIF]

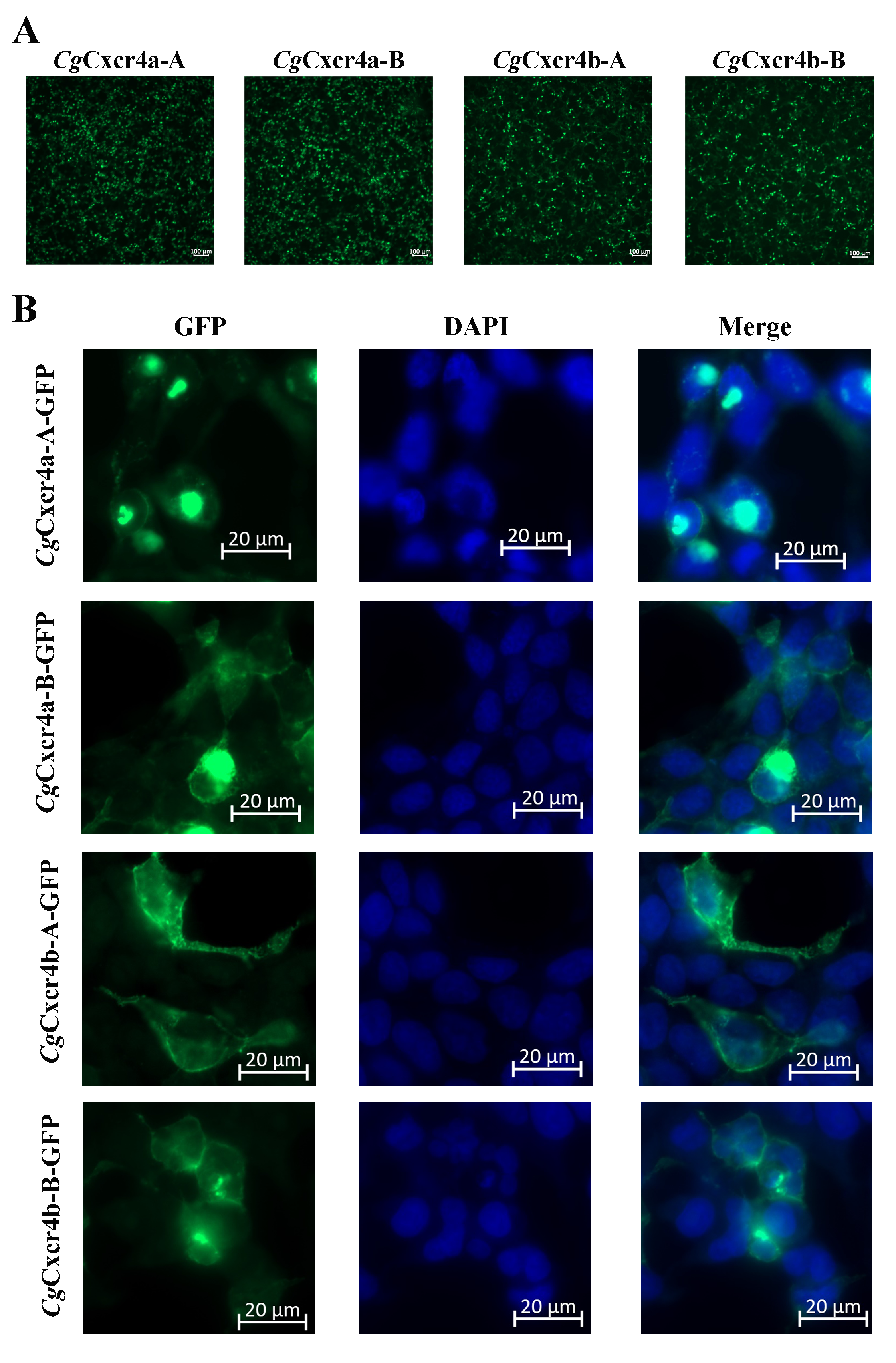

Supplement: Supplementary Figure 5 — The transfection efficiency and subcellular localization of CgCxcr4s. [file Image_5.TIF]

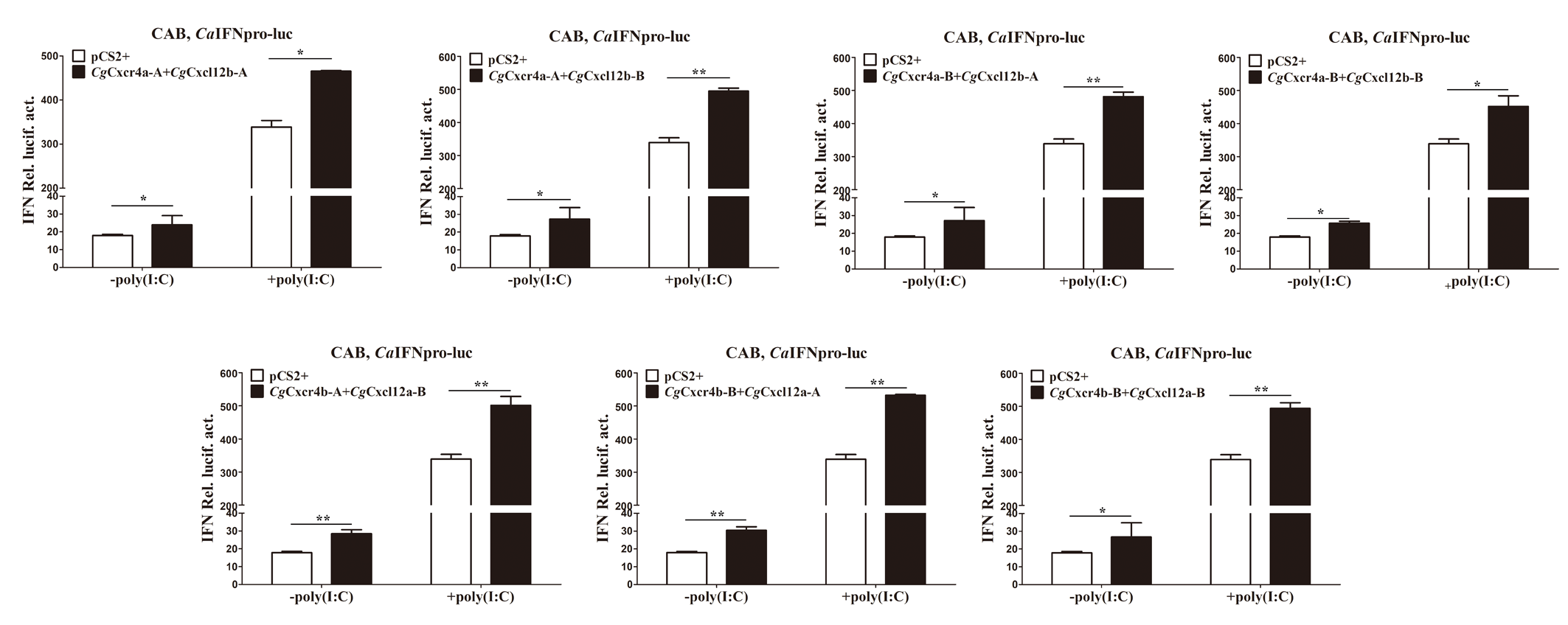

Supplement: Supplementary Figure 6 — Different CgCxcl12 and CgCxcr4 combinations activated the activity of CaIFN promoter. [file Image_6.TIF]
